# HOXD10 promotes hepatocellular carcinoma progression by enhancing m6A methylation and activating the PI3K/AKT/mTOR pathway

**DOI:** 10.1186/s12967-026-08632-x

**Published:** 2026-07-13

**Authors:** Chengdong Qin, Xinyi Gao, Siyuan Liu, Jiejie Hu, Lihong Hu, Chao Hu, Yang Yu, Dening Ma

**Affiliations:** 1 Department of Breast Surgery, Zhejiang Cancer Hospital, Hangzhou, Zhejiang Province China; 2 Department of Radiology, Zhejiang Cancer Hospital, Hangzhou, Zhejiang Province China; 3 Postgraduate Training Base Alliance of Wenzhou Medical University (Zhejiang Cancer Hospital), Hangzhou, Zhejiang Province 310022 China; 4 Department of Hepatobiliary and Pancreatic Surgery, Zhejiang Cancer Hospital, Hangzhou, Zhejiang Province China

**Keywords:** HOXD10, Hepatocellular carcinoma, Prognosis, Hepatic cancer stem cells, m6A methylation

## Abstract

**Background:**

The Homeobox (HOX) gene family is capable of binding to specific deoxyribonucleic acid (DNA) sequences, thereby modulating gene expression through activation or repression, which subsequently influences cellular behaviors including proliferation, adhesion, differentiation, and metastasis. Nevertheless, the role of Homeobox D10 (HOXD10) in the prognosis and progression of hepatocellular carcinoma (HCC) remains to be elucidated.

**Methods:**

The expression of HOXD10 in HCC was subsequently validated through Western blot and immunohistochemistry analysis. The functional role of HOXD10 in HCC was evaluated using a combination of high- and low-expression experiments, incorporating both in vitro and in vivo methodologies. Furthermore, The Cancer Genome Atlas (TCGA) database was employed to investigate the functional networks related to HOXD10. The research specifically investigated the relationship between HOXD10 expression and cancer stem cells (CSCs), emphasizing the role of N6-methyladenosine (m6A) methylation and the PI3K/AKT/mTOR signaling pathway in hepatocellular carcinoma (HCC).

**Results:**

The marked upregulation of HOXD10 expression in HCC suggests its potential impact on adverse prognostic outcomes. Furthermore, HOXD10 expression exhibited a significant association with the T stage, vascular invasion, and histological grade of HCC. Elevated HOXD10 expression was found to significantly enhance HCC cell proliferation, invasion, and migration. Notably, there was a significant correlation between HOXD10 expression and the activation of m6A methylation, as well as the PI3K/AKT/mTOR signaling pathway.

**Conclusion:**

The results of this study corroborate the hypothesis that HOXD10 serves as a novel biomarker for tumor progression in HCC. Furthermore, HOXD10 has the capacity to activate the PI3K/AKT/mTOR signaling pathway through the regulation of m6A RNA methylation levels, thereby facilitating the self-renewal of hepatic cancer stem cells (HCSCs).

**Supplementary Information:**

The online version contains supplementary material available at https://doi.org/10.1186/s12967-026-08632-x.

## Background

Liver cancer, primarily hepatocellular carcinoma (HCC), is one of the most aggressive cancers, accounting for over 90% of liver cancer cases. In 2022, HCC was the seventh most common cancer globally, with 870,000 new cases and 760,000 deaths, making it the third leading cause of cancer-related deaths [1]. Its mortality rate closely matches its incidence rate, indicating a poor prognosis. The unfavorable clinical outcomes associated with hepatocellular carcinoma (HCC) are primarily due to delayed diagnosis, high rates of postoperative recurrence, early vascular invasion, and extensive resistance to existing therapeutic interventions [2]. From an epidemiological perspective, chronic infections with hepatitis B virus (HBV) and hepatitis C virus (HCV), along with liver cirrhosis, alcohol abuse, obesity, and metabolic-associated fatty liver disease, represent significant risk factors [2, 3]. These factors exhibit distinct geographic distributions across Asia, Africa, and Western nations [4]. Despite progress in treatments like surgery, transplantation, and targeted therapies, long-term survival rates are still poor, highlighting the need for new molecular research and more effective treatments.

About 25–50% of liver cancer patients show vascular invasion, which is linked to multiple tumors, larger tumor sizes, and high alpha-fetoprotein (AFP) levels, indicating a high recurrence risk [5]. Optimal treatment for these patients involves wider surgical margins or anatomical liver resection. Patients outside the Milan criteria but without vascular invasion can have a 65.8% five-year survival rate [6]. Angiolymphatic invasion is a more significant predictor of outcomes than tumor size or number. Current markers like AFP and des‑γ‑carboxy prothrombin (DCP) do not indicate vascular invasion, and liver cancer’s imaging traits often make preoperative biopsy unnecessary, complicating the assessment of vascular invasion. Identifying biomarkers specific to vascular invasion is crucial for accurately assessing its presence at diagnosis and guiding treatment plans. Additionally, discovering genes that drive vascular invasion offers new therapeutic targets, potentially enhancing survival rates in HCC patients.

Despite significant progress in HCC treatment, current therapies are limited by poor targeting, toxicity, and resistance. Fortunately, rapid advances in interdisciplinary technologies have created new opportunities for enhancing HCC therapy. Micro-nano delivery systems, such as microrobots, offer precise targeting and efficient drug delivery, thereby enhancing treatment efficacy and reducing side effects [7]. The food and medicine homology concept provides a low-toxicity approach for synergistic treatment and immune regulation [8]. Additionally, altering cancer cells’ physical properties presents a novel intervention strategy [9]. Despite these advances, the molecular mechanisms driving HCC progression, stemness, and treatment resistance are not fully understood, highlighting the need for detailed study of key regulatory molecules and signaling networks.

Cancer Stem Cells (CSCs) are a unique group of tumor cells with malignant traits and stem-like abilities, such as self-renewal, differentiation, and therapy resistance [10]. Hepatic cancer stem cells (HCSCs), found at the invasive edge of liver tumors, are crucial for metastasis [11]. These cells promote new blood vessel growth via Vascular Endothelial Growth Factor (VEGF) secretion, impacting vessel integrity and aiding tumor spread [12]. Besides traditional angiogenesis, vasculogenic mimicry (VM) allows cancer cells to form vessel-like structures, ensuring blood supply for tumor growth and metastasis. VM is a key reason for the failure of current angiogenesis inhibitors in treating HCC [13]. Recent studies link HCSCs to VM formation, with research showing that lncRNA n339260 promotes VM in HCC by inducing CSCs [14]. HCSCs secrete angiogenic factors that enhance angiogenesis and confer resistance to anti-angiogenic therapies in HCC via VM. The PI3K/AKT/mTOR pathway activation increases hypoxia-inducible factor 1α (HIF-1α) and VEGF expression, boosting vascular permeability and facilitating tumor cell invasion into the bloodstream [15]. Transcriptomic analysis of 3,000 tumor samples shows a strong correlation between PI3K/AKT/mTOR activity and tumor stemness [16]. Thus, effective anti-angiogenic therapies for HCC should target CSCs.

The HOX gene family features a conserved 183-base pair homeobox domain encoding a 61-amino-acid helix-turn-helix motif that binds specific DNA sequences to regulate gene expression and cellular behaviors such as proliferation and metastasis [17]. Aberrant HOX gene expression is linked to tumor mechanisms, including spatial-temporal heterogeneity and epigenetics [18]. Homeobox D10 (HOXD10), a HOX family member, shows abnormal expression in various tumors. In colorectal cancer, high HOXD10 levels correlate with poor outcomes; while in ovarian cancer, they inhibit metastasis. These findings highlight the varied roles of HOXD10 across different tumors and confirm the crucial role of HOX genes in stem cell differentiation [19]. S. Bhatlekar et al. [20] found higher HOXD10 expression in colonic stem cells than in other crypt cells. The role of HOX genes in liver cancer is still unclear and needs more research.

Epigenetics mediates interactions between tumor cells and their microenvironment, playing a crucial role in invasion and metastasis by swiftly altering gene expression and cell phenotype [21]. Research has mainly focused on transcriptional levels, especially reversible RNA methylation, with N6-methyladenosine (m6A) being the most common mRNA methylation in mammals. This modification influences gene expression by affecting mRNA stability, splicing, transport, localization, translation, and RNA interactions [22]. Recent studies highlight m6A’s role in oncogenic processes like tumor initiation and metastasis [23]. Notably, HOX genes regulate methyltransferase like 3 (METTL3) expression, impacting m6A modification and promoting gastric cancer metastasis [24].

This study examines the regulatory role of HOXD10 in hepatocellular carcinoma (HCC), focusing on its effects on hepatic cancer stem cells (HCSCs). We hypothesize that HOXD10 influences m6A RNA methylation via METTL3 and YTH N6‑methyladenosine RNA binding protein 1 (YTHDF1) and activates the PI3K/AKT/mTOR pathway. By exploring these mechanisms, we aim to offer new insights into epigenetic regulation in HCC and identify potential therapeutic targets.

## Methods

### HCC samples collection

This study was approved by the Clinical Research Ethics Committee of Zhejiang Cancer Hospital. Written informed consent was obtained from each patient prior to tissue collection in accordance with the committee’s policies. HCC tissue samples and paired non-tumor tissue samples were used for Western blot and immunohistochemistry staining.

### Tumor database analysis

Gene expression data (RNA-seq via STAR pipeline) and clinical details for hepatocellular carcinoma were sourced from the TCGA-LIHC project via the Genomic Data Commons (GDC) Data Portal (https://portal.gdc.cancer.gov). Normal liver tissues and incomplete clinical cases were excluded, leaving 374 patients for analysis. Gene expression was measured in transcripts per million (TPM) and fragments per kilobase million (FPKM), normalized with log2(value+1). Patients were categorized into high and low HOXD10 expression groups using the median expression value as a cutoff. Using the clusterProfiler tool, Gene Ontology (GO) and Kyoto Encyclopedia of Genes and Genomes (KEGG) pathway enrichment analyses were carried out on protein-coding gene (PCGs) associated with HOXD10 [25]. Enrichment results were depicted in R through the ggplot2 package.

### Lentivirus construction and cell culture

Lentivirus-mediated short hairpin RNA (shRNA) targeting Homeobox Protein Hox-D10 (HOXD10, NCBI Gene: 3236) was purchased from Zorin Bio-Company (Shanghai, China), and inserted into the lentiviral vector pGMLV-SC5 RNAi for the expression of HOXD10 knockdown shRNA. Similarly, the target gene was cloned into the PGMLV-CMV-MCS-3×Flag-EF1-ZsGreen1-T2A-Puro vector for the construction of the HOXD10 overexpression lentiviral vector.

Huh7 and HCCLM3 cell lines were chosen to model HCC progression: Huh7 for early-stage, well-differentiated HCC, and HCCLM3 for advanced, poorly differentiated, highly metastatic HCC, enabling thorough validation of malignant traits at various stages. Cells were cultured in Dulbecco’s Modified Eagle’s Medium (DMEM; Catalog No.: L110KJ; Basal Media, Shanghai, China) with 10% fetal bovine serum (FBS; Catalog No.: 10100147C; Gibco, Grand Island, NY), 100 units/ml penicillin, and 100 μg/mL streptomycin added. Huh7 cells were infected with an appropriate amount of virus in the presence of polybrene at a final concentration of 10 µg/mL. Following 48 hours of infection, stable cell lines were established through selection with puromycin for subsequent experiments. A similar protocol was employed to generate an HCCLM3 cell line with reduced HOXD10 expression.

### In vitro cell behavior assays

Cell proliferation assay: Cell proliferation was assessed using the Cell Counting Kit-8 (CCK-8; Catalog No.: C6050; NCM Biotechnology, China). Specifically, 10 µL of CCK-8 reagent was added to each well, followed by a 2-hour incubation. Absorbance at 450 nm was then measured to evaluate cell proliferation.

Colony formation assay: Cells were digested, resuspended, and seeded at a density of 1,000 cells per well in a 6-well plate. The cells were incubated for up to 14 days or until individual colonies contained more than 50 cells. Colonies were then stained with crystal violet, and images were captured for analysis.

5-ethynyl-2′-deoxyuridine (EdU) incorporation assay: Cell proliferation was further assessed using an EdU assay kit (Catalog No.: C0076L; Beyotime, Haimen, China). HCC cells were seeded at a density of 1 × 10^4^ cells per well in a glass-bottom dish and incubated overnight. The cells were then incubated with a 10 µM EdU solution at 37 °C for 2 hours, followed by fixation with 4% formaldehyde for 15 minutes and permeabilization with 0.3% Triton X-100 for 25 minutes. Hoechst staining was used for nuclear visualization. EdU-positive cells (red fluorescence) were observed under a fluorescence microscope, and EdU incorporation rate was assessed by the ratio of cells positive for EdU (red) to the total cells positive for Hoechst (blue).

Transwell Assay: Cell invasion capacity was assessed using a Transwell assay (Catalog No.: 3464; Boyden Chambers; Corning Inc.). After digestion, cells were resuspended in a serum-free medium, adjusting the density to 2 × 10^5^ cells/mL. Then, 700 µL of culture medium containing 10% FBS was added to the lower chamber of a 24-well plate, and 500 µL of the cell suspension was placed in the upper chamber. Following a 24-hour incubation, cells that traversed the membrane were stained with crystal violet, and representative images were captured for analysis.

Wound healing assay: Cells were plated evenly in a 6-well plate, and a scratch was created in the central region of the cell monolayer using a 10-µL pipette tip. Images were captured at 0 and 48 hours, and the width of the scratch was measured at regular intervals to evaluate the rate of cell migration during the wound healing process.

Stem Cell Sphere Formation Suspension Assay: Cells were digested, centrifuged, and washed with phosphate-buffered saline (PBS) to remove the serum-containing medium. Subsequently, the cells were resuspended in a stem cell culture medium comprising DMEM/F12 supplemented with 1× B27, 20 ng/mL basic fibroblast growth factor (bFGF), and 20 ng/mL epidermal growth factor (EGF), followed by cell counting. Ultra-low attachment six-well plates (Catalog No.: 3471; Boyden Chambers; Corning Inc.) were used for sphere formation, with each well seeded with an appropriate number of cells (1,000 cells per well). After approximately two weeks, the culture was completed. The spheres formed during the first round of culture were collected, and cells were replated for a second round of culture following the same procedure. Sphere formation was evaluated and photographed after another two weeks.

### Immunohistochemistry (IHC) staining

Tissue sections (5 µm) were deparaffinized with dimethylbenzene and rehydrated through graded ethanol. Antigen retrieval was performed in 10 mM citrate buffer (pH 6.0) by microwaving at 100 °C for 20 minutes. Sections were first incubated with an endogenous peroxide block and then treated with 5% bovine serum albumin (BSA) for blocking (Catalog No.: ST023; Beyotime Biotechnology, China), followed by the overnight incubation at 4 °C with the primary antibody. The 3,3′-diaminobenzidine (DAB) IHC detection kit was employed for IHC staining (Catalog No.: PV-9000; ZSGB‑BIO, Beijing, China). To calculate the IHC score, four random fields (magnification 200×) were selected from each slide, and the integrated optical density (IOD) of each image was measured using Image-Pro Plus 6.0 software. The final IHC score was represented as the average IOD from the four images. IHC was performed on human primary HCC samples and corresponding adjacent normal tissue samples using the HOXD10 specific antibody.

### RNA dot blot

RNA samples were extracted, quantified, and exposed to 95 °C for a duration of 3 minutes. A 2 μL sample was applied onto a nylon membrane, which was then oven-dried at 37 °C for 30 minutes. The membrane was washed with TBST and blocked for 1 hour before being incubated with the primary antibody overnight at 4 °C. After three times of TBST-washing, a secondary antibody was applied for 1 hour. Detection was performed using an ECL substrate (Catalog No.:P10200; NCM Biotechnology, China) for 5 minutes, after which the membrane was imaged. The membrane was then stained with methylene blue for 30 minutes, rinsed with ddH₂O, and imaged under white light.

### Western blotting

Cells were lysed in RIPA buffer (Catalog No.: WB3100; NCM Biotechnology, China) containing protease and phosphatase inhibitors, with all procedures maintained on ice. After separation by sodium dodecyl sulfate-polyacrylamide gel electrophoresis (SDS-PAGE), proteins were transferred to polyvinylidene fluoride (PVDF) membranes (Catalog No.: 3010040001; Millipore, Bedford, MA) and blocked with 5% milk for one hour. To account for the significant discrepancies in molecular weights between the target proteins and the loading controls, the membrane was segmented according to the pre-stained molecular weight markers before proceeding with antibody incubation. Overnight incubation of membranes at 4 °C with primary antibodies was followed by washing with Tris-buffered saline with Tween 20 (TBST) and a one-hour treatment with horseradish peroxidase (HRP)-conjugated secondary antibodies at room temperature. Protein bands were visualized using an enhanced chemiluminescence (ECL) kit (Catalog No.:P10200; NCM Biotechnology, China) and analyzed with Quantity One software. The antibodies utilized in this study were procured from ZENBIO, China.

### Xenograft model of human HCC in nude mice

Male BALB/c nude mice from Shanghai SLAC Laboratory Animal Co. Ltd. were raised in pathogen-free conditions. For the xenograft model, 5 × 10^6^ Huh7 and HCCLM3 cells were injected subcutaneously into the left upper flank of three 4-week-old mice. After one month, the tumors were excised, sectioned into 1 mm^3^ fragments, and subsequently implanted into the liver’s left lobe (five mice per group) [26]. Thirty days post-implantation, mice were euthanized, and tumors were harvested, photographed, and weighed. Tumor volume was calculated using the following formula: (maximum diameter × minimum diameter^2^)/2. All procedures were conducted in accordance with the guidelines of the Laboratory Animal Care Committee’s guidelines.

### Ethical approval, euthanasia, and anesthesia

This study received approval from the Animal Ethics Committee of Zhejiang Cancer Hospital (2022–02-080). Euthanasia of nude mice was performed by cervical dislocation. For anesthesia, sodium pentobarbital (25 mg/kg body weight) was administered intraperitoneally.

### Statistical analysis

Data are presented as mean ± Standard Deviation (SD) from at least three independent experiments and analyzed using SPSS 26.0 and GraphPad Prism 9.0. Differences between two groups were assessed with the Student’s t-test, while one-way analysis of variance (ANOVA) with Tukey’s multiple comparisons test or two-way ANOVA with Dunnett’s multiple comparisons test was used for comparisons involving more than two groups. The χ^2^ test and Spearman correlation test examined correlations between HOXD10 expression and clinicopathological features in HCC patients. Kaplan–Meier method and log-rank test were used for overall and disease-free survival curves. Prognostic factors were identified through univariate and multivariate Cox regression analyses. For the in vivo xenograft experiments, differences in tumor volume and lung metastatic nodule numbers were analyzed by the Student’s t-test. Statistical significance was defined as two-tailed *p* < 0.05.

### Ethics statement for human research

Any studies utilizing human subjects, human-derived samples, or human data complied strictly with the ethical principles established by the World Medical Association’s Declaration of Helsinki (https://www.wma.net/policies-post/wma-declaration-of-helsinki-ethical-principles-for-medical-research-involving-human-subjects/).

The study adhered to the ARRIVE Guidelines for Reporting Research (see https://pubmed.ncbi.nlm.nih.gov/32663219/ for reference).

## Results

### HOXD10 was aberrantly elevated in HCC and correlated with poor prognosis

**Fig. 1:**
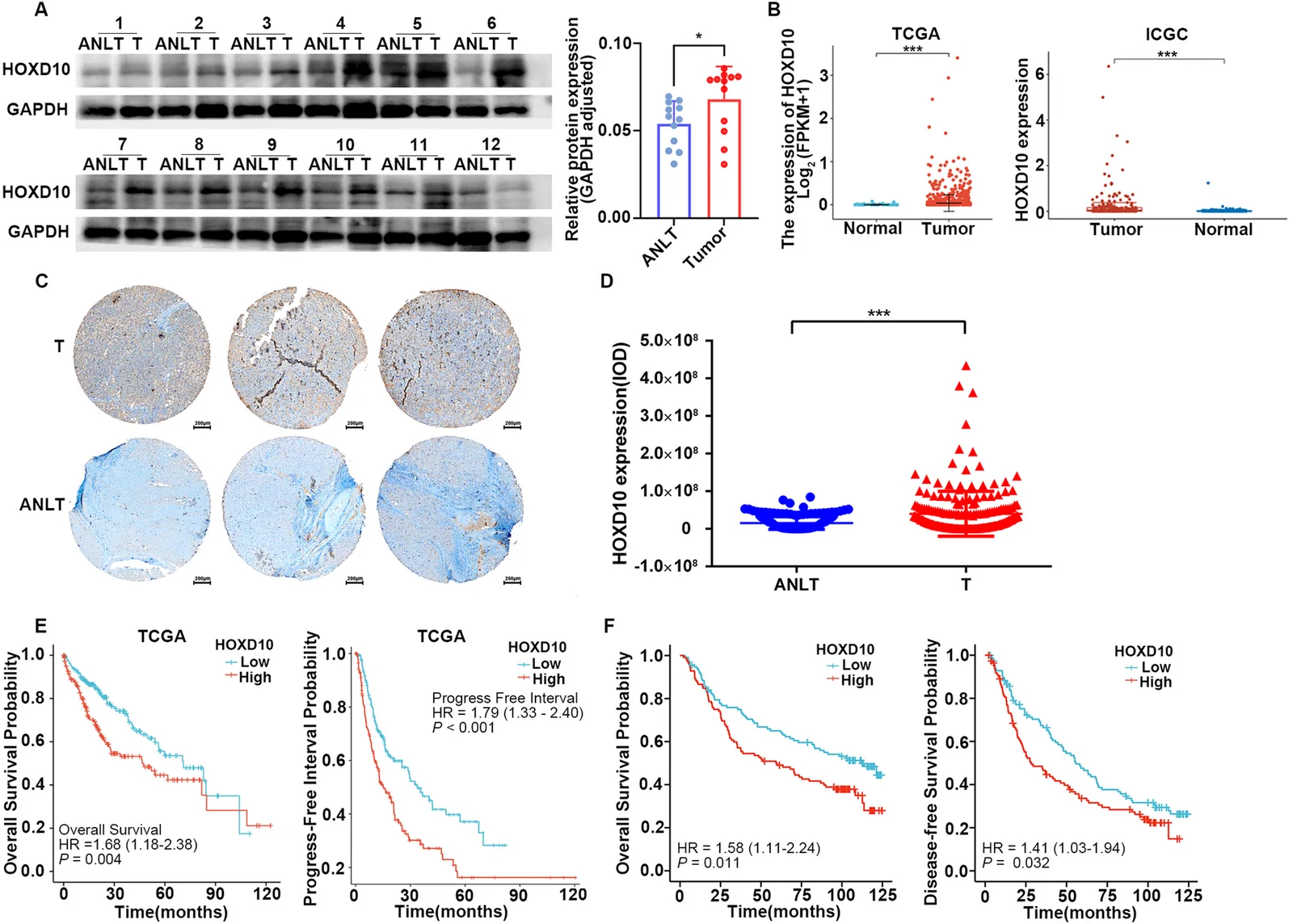
HOXD10 expression in HCC tissues. (**A**) Western blot analysis of the differential expression of HOXD10 protein in HCC tissues (T; *n* = 12) and corresponding adjacent non-cancerous tissues (ANLTs; *n* = 12). Densitometry of western blot in panel. Expression level of HOXD10 was normalized to GAPDH. Significant difference was tested by Student’s t-test. *p < 0.05. (**B**) Differential expression of HOXD10 protein in HCC tissues and corresponding adjacent non-cancerous tissues from TCGA (Normal = 50; Tumor = 374) and ICGC database (Normal = 202; Tumor = 240), significant difference was tested by Student’s t-test. ***p < 0.001. (**C**, **D**) Representative IHC images of HOXD10 expression in HCC tissues (T; *n* = 225) and adjacent nontumor liver tissues (ANLTs; *n* = 227). Scale bar: 200 μm. HOXD10 expression was quantitated based on integrated optical density (IOD). Results were expressed as mean ± SD. Significant difference was tested by Student’s t-test. ***p < 0.001. (**E**) Survival analysis based on the TCGA database. (Left) Overall survival (OS) and (Right) Progression-free interval (PFI) of HCC patients stratified by HOXD10 expression levels. The median expression value of HOXD10 was used as the cutoff to divide patients into high (red line; *n* = 186) and low (blue line; *n* = 187) expression groups. (**F**) The Kaplan-Meier survival analysis was conducted on the in-house hepatocellular carcinoma (HCC) cohort. The analysis examined (left) overall survival (OS) and (right) disease-free survival (DFS) among patients categorized according to HOXD10 expression levels. Patients were stratified into high (red line; *n* = 112) and low (blue line; *n* = 113) HOXD10 expression groups, using the median expression value as the cutoff point

To evaluate the expression of HOXD10 in HCC patients, we analyzed cancerous and adjacent non-cancerous tissue samples from 12 matched pairs of HCC patients. Western blot analysis indicated a significant elevation in HOXD10 protein expression in liver cancer tissues compared to adjacent non-cancerous tissues (Fig. 1A). Consistent with these results, data from the TCGA and the International Cancer Genome Consortium (ICGC) databases also demonstrated increased HOXD10 expression in HCC tissues relative to normal tissues, which correlated with a less favorable prognosis in patients (Fig. 1B, E). Additionally, IHC analysis demonstrated that HOXD10 was markedly overexpressed in HCC tissues (Fig. 1C, D). Based on the IOD values obtained from IHC staining, patients were stratified into high and low HOXD10 expression groups. The follow-up results suggested that patients with high HOXD10 expression had significantly diminished overall survival (OS) and disease-free survival (DFS) (Fig. 1F). Using TCGA data, multivariate Cox regression analysis revealed that high HOXD10 expression is an independent risk factor for shorter OS and shorter progression-free interval (PFI) in HCC patients, even after adjusting for factors like T stage, vascular invasion, and distant metastasis (Supplementary Tables 1 and 2). In conclusion, increased HOXD10 expression in HCC patients was associated with unfavorable prognosis.

### HOXD10 expression correlated with tumor stage and vascular invasion in HCC

**Fig. 2:**
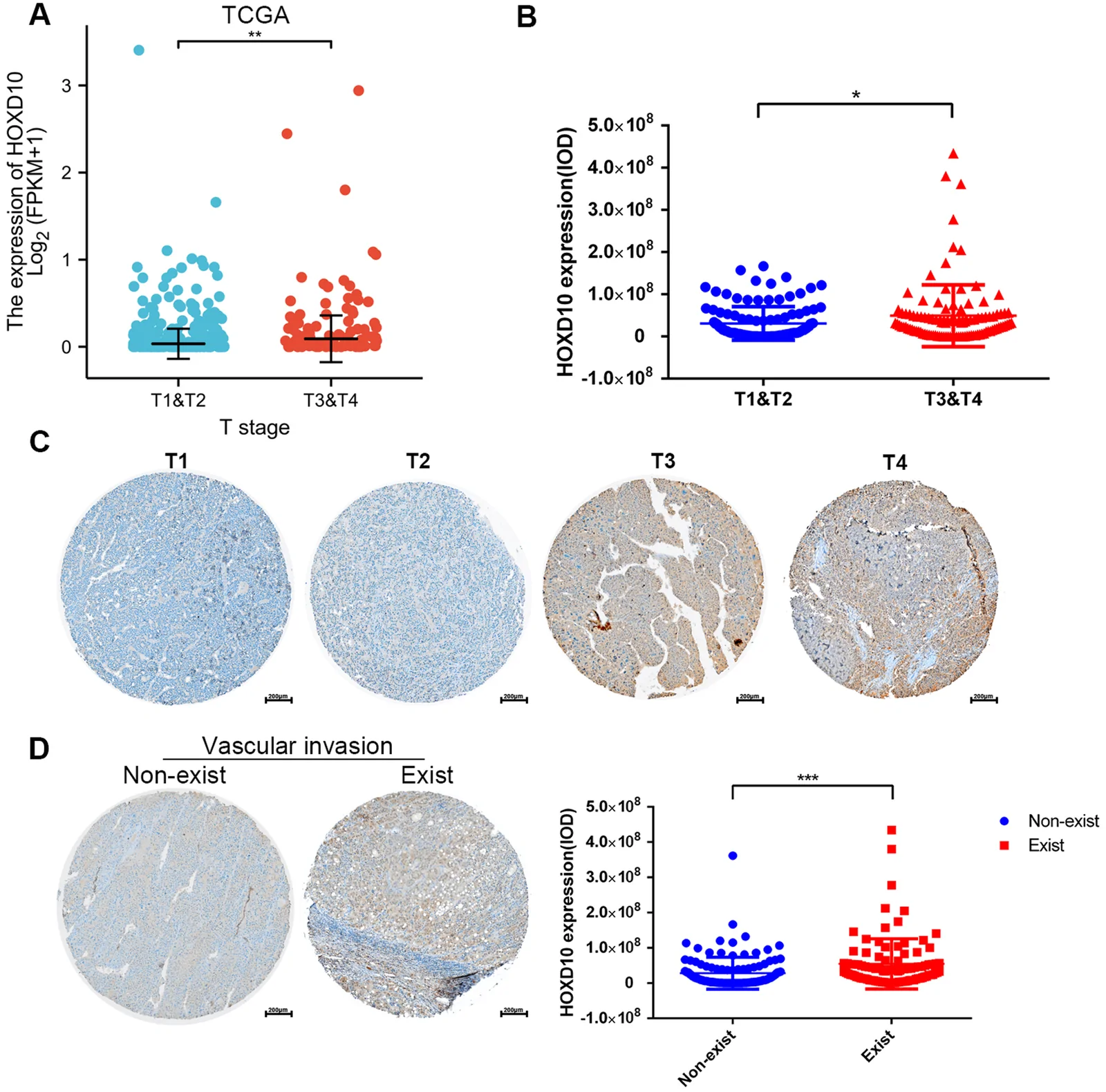
HOXD10 expression in relation to tumor stage and vascular invasion in HCC. (**A**) HOXD10 expression levels in HCC patients across different T stages in the TCGA cohort were compared between T1&T2 (blue) and T3&T4 (red) groups. Data are shown as median with interquartile range, and significant differences were assessed using Student’s t-test (**p < 0.01). (**B**) Immunohistochemical analysis measured HOXD10 expression in HCC patients by T stage, using IOD. T1&T2 stages are shown as blue circles and T3&T4 as red triangles, with data presented as median and interquartile range. Significant differences were determined by Student’s t-test, *p < 0.05. (**C**) Representative immunohistochemical staining of HOXD10 in HCC tissues with different T stages (T1, T2, T3, T4). Scale bar: 200 μm. (**D**) Immunohistochemical analysis of HOXD10 in HCC tissues based on vascular invasion status. Representative IHC staining shows HOXD10 in tissues with and without vascular invasion. Scale bar: 200 μm. HOXD10 expression quantified by IOD, comparing patients with (red squares) and without (blue circles) vascular invasion, presented as median with interquartile range. Significant differences were determined by Student’s t-test, ***p < 0.001

To further elucidate the relationship between HOXD10 expression and clinical pathological features, we conducted a correlation analysis utilizing TCGA database. The findings indicated a significant association between HOXD10 expression and tumor stage (T-stage), pathologic stage as well as vascular invasion (Table 1). An analysis of the TCGA database revealed that the expression level of HOXD10 was significantly elevated in the T3 and T4 groups compared to the T1 and T2 groups (Fig. 2A). This finding was further substantiated by IHC analysis, which demonstrated a marked increase in HOXD10 expression in the T3 and T4 stages relative to the T1 and T2 stages (Fig. 2B, C). Furthermore, HOXD10 expression was significantly higher in the vascular invasion groups compared to the non-vascular invasion groups, with statistically significant differences observed (Fig. 2D). These results implied that HOXD10 may play a role in both the proliferation and metastasis of HCC.

**Table 1:** HOXD10 is associated with poor pathological features

Characteristic	HOXD10-Low	HOXD10-High	p
n	187	187	
T stage, n (%)			**0.001**
T1	109 (29.4%)	74 (19.9%)	
T2	39 (10.5%)	56 (15.1%)	
T3	33 (8.9%)	47 (12.7%)	
T4	3 (0.8%)	10 (2.7%)	
Pathologic stage, n (%)			**<0.001**
Stage I	103 (29.4%)	70 (20%)	
Stage II	34 (9.7%)	53 (15.1%)	
Stage III	32 (9.1%)	53 (15.1%)	
Stage IV	3 (0.9%)	2 (0.6%)	
Vascular invasion, n (%)			**0.003**
No	123 (38.7%)	85 (26.7%)	
Yes	45 (14.2%)	65 (20.4%)

### HOXD10 promoted proliferation, invasion and migration of HCC cells

**Fig. 3:**
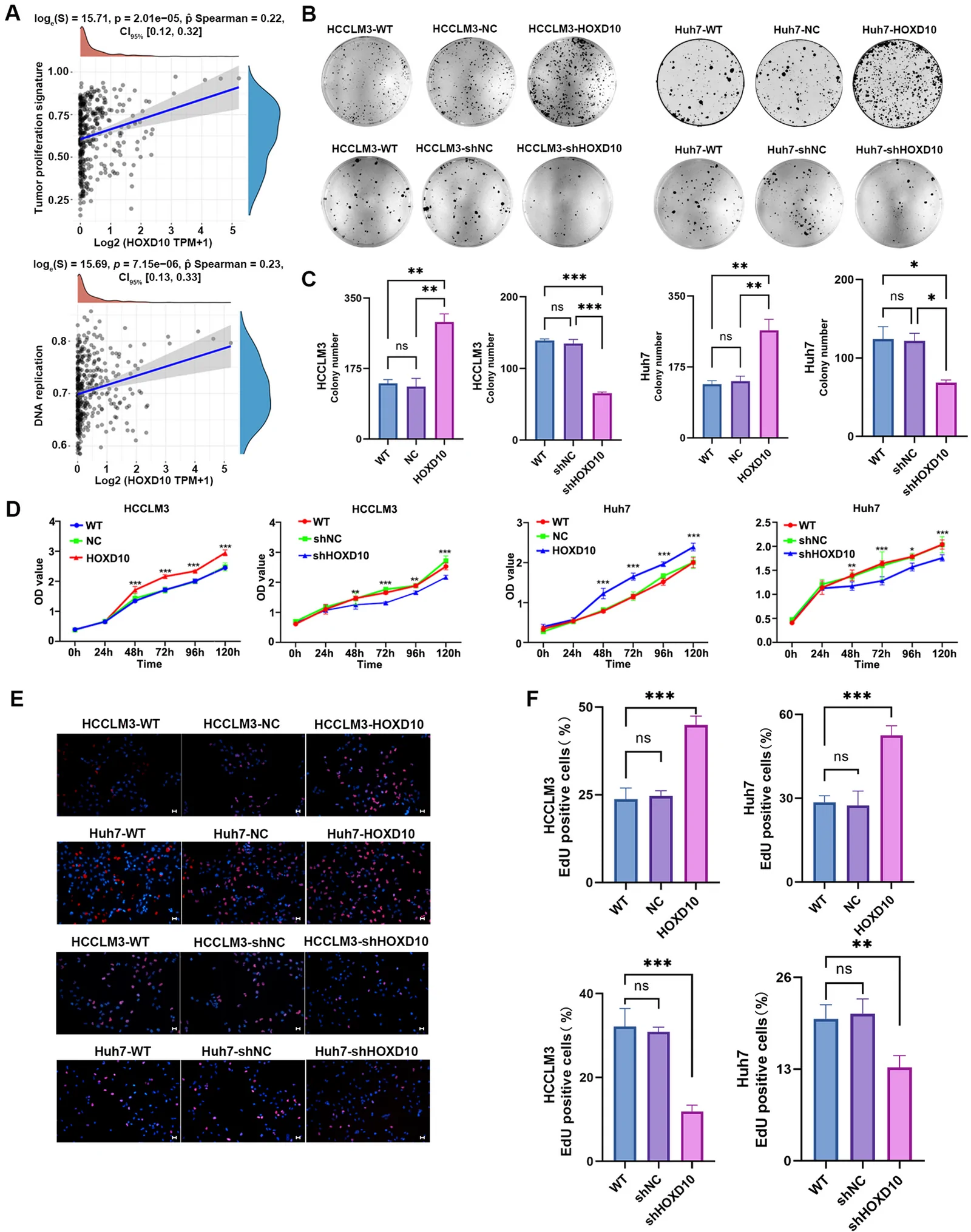
HOXD10 promotes proliferation of HCC cells. (**A**) The ssGSEA algorithm results indicate that HOXD10 expression is positively correlated with the tumor proliferation signature and DNA replication signaling pathways in HCC. (**B**, **C**) Colony formation assay to evaluate the impact of HOXD10 on the proliferation capacity of HCC cells. The number of colonies was counted in triplicate plate and compared in the diagrams, significance was determined using one-way ANOVA with Tukey’s multiple comparisons test. *p < 0.05, **p < 0.01, ***p＜0.001. (**D**) CCK8 assay confirming the effect of HOXD10 on the proliferation rate of HCC cells. All values are expressed as mean ± SD (*n* = 4), significance was determined using two-way ANOVA with Dunnett’s multiple comparisons test. *p < 0.05, **p < 0.01, ***p＜0.001. (**E**) EdU incorporation assay assessing the effect of altered HOXD10 expression on the proliferation of HCC cells. Representative EdU staining images of HCCLM3 and Huh7 cells after HOXD10 overexpression or knockdown. Scale bar: 50 μm. (**F**) Quantification of EdU-positive cells in HCCLM3 and Huh7 cells. Data are presented as mean ± SD (*n* = 3) of three independent experiments, significant difference was tested by one-way ANOVA with Tukey’s multiple comparisons test. * *p* < 0.05, **p < 0.01, ***p < 0.001, ns, not significant

To investigate the impact of HOXD10 on the biological behavior of HCC cells, we first conducted a correlation analysis utilizing the TCGA database and the Gene Set Enrichment Analysis (ssGSEA) algorithm. The analysis revealed a robust positive correlation between HOXD10 expression and tumor proliferation signatures, as well as DNA replication signaling pathways (Fig. 3A). These findings hinted that HOXD10 may play a role in modulating HCC cell proliferation. Subsequently, we established HCC cell lines with either overexpression (HCCLM3–HOXD10, Huh7-HOXD10) or knockdown (HCCLM3-shHOXD10, Huh7-shHOXD10) of HOXD10 through lentiviral transfection. Results from CCK8 and colony formation assays demonstrated that overexpression of HOXD10 enhanced HCC cell proliferation, whereas knockdown of HOXD10 led to a reduction in proliferation (Fig. 3B–D). These observations were further corroborated by EdU incorporation assays, which showed a significant increase in EdU-positive cells in the HOXD10 overexpression group compared to controls, with the inverse pattern observed in the knockdown group (Fig. 3E, F). Moreover, we explored the potential role of HOXD10 in HCC cell migration and invasion. Scratch and transwell assays demonstrated that elevated HOXD10 expression considerably enhanced the migratory and invasive capacities of HCC cells, while reduced HOXD10 expression inhibited these capabilities (Supplementary Fig. 1A–D). Collectively, these results indicated that HOXD10 upregulation was critical for facilitating HCC cell proliferation, migration and invasion.

### HOXD10 overexpression promotes HCC growth in vivo

**Fig. 4:**
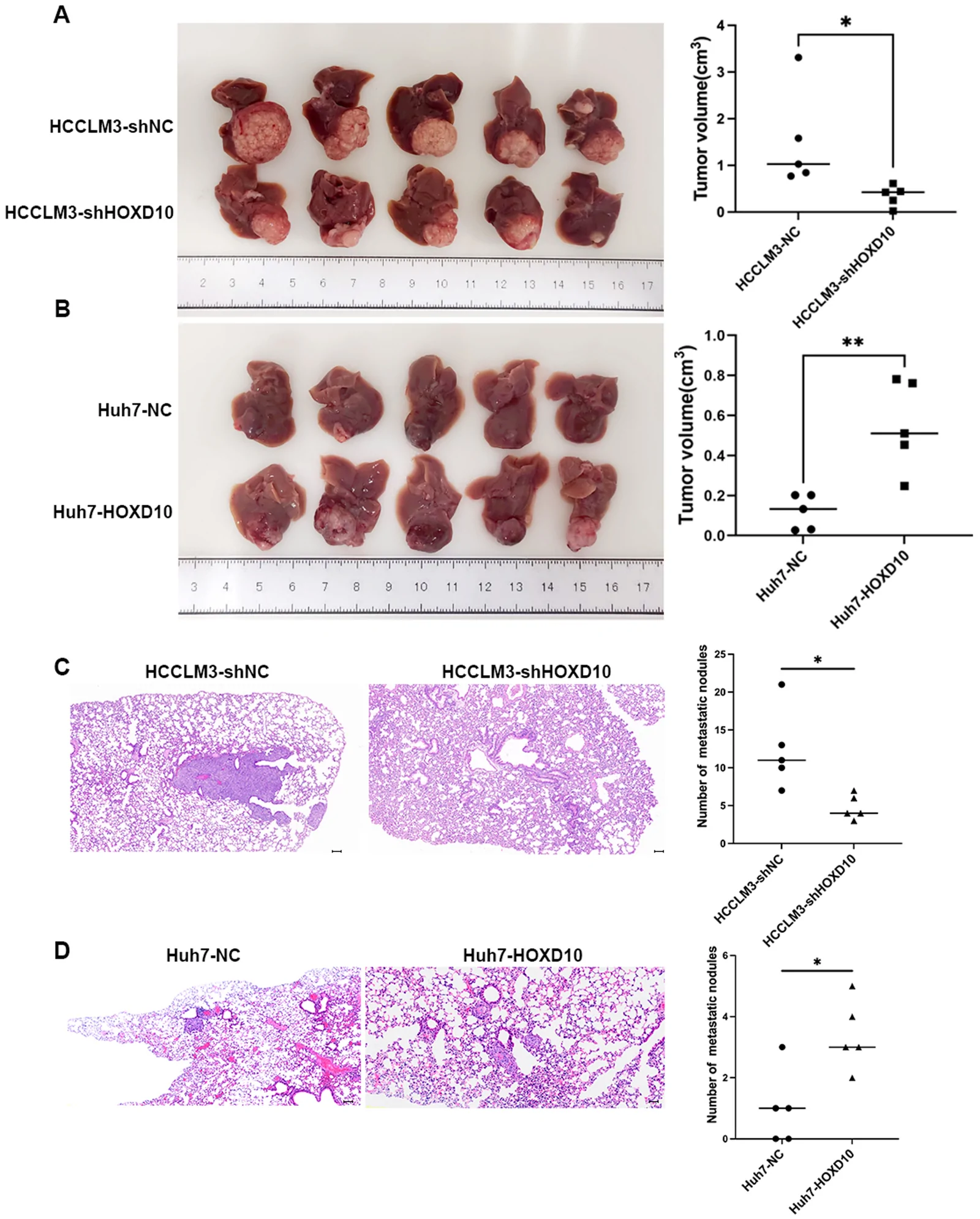
HOXD10 overexpression promotes HCC growth in vivo. (**A**, **B**) Orthotopic liver cancer model in nude mice (5 mice per group) to assess the impact of HOXD10 expression on HCC growth in vivo. The size of liver tumors in each group was calculated and compared. The volume of tumors in each group was calculated with formula: (Length×Width^2^)/2. Significant difference was tested by Student’s t-test. * *p* < 0.05, **p＜0.01. (**C**, **D**) Representative HE staining images for lung metastasis. Scale bar: 100 μm. The number of lung metastatic nodules in each group of mice (5 mice per group) was calculated and compared. Significant difference was tested by Student’s t-test. *p < 0.05

To investigate the role of HOXD10 in tumor progression in vivo, we conducted an orthotopic tumor formation experiment. Approximately one month following tumor development, livers and lungs were harvested from each group of mice to assess tumor volume and evaluate the tumorigenic capacity of the different cell lines. Additionally, the number of lung metastases were quantified to determine metastatic potential. The findings indicated that the downregulation of HOXD10 expression in HCCLM3 cells was associated with reduced tumor volume and a lower incidence of lung metastasis. Conversely, the overexpression of HOXD10 in Huh7 cells resulted in an increase in both tumor volume and the number of lung metastases (Fig. 4A–D). These findings underscored the pivotal role of HOXD10 in promoting liver tumor development and facilitating lung metastasis in HCC.

### HOXD10 regulates cancer stem cell-like properties in HCC

**Fig. 5:**
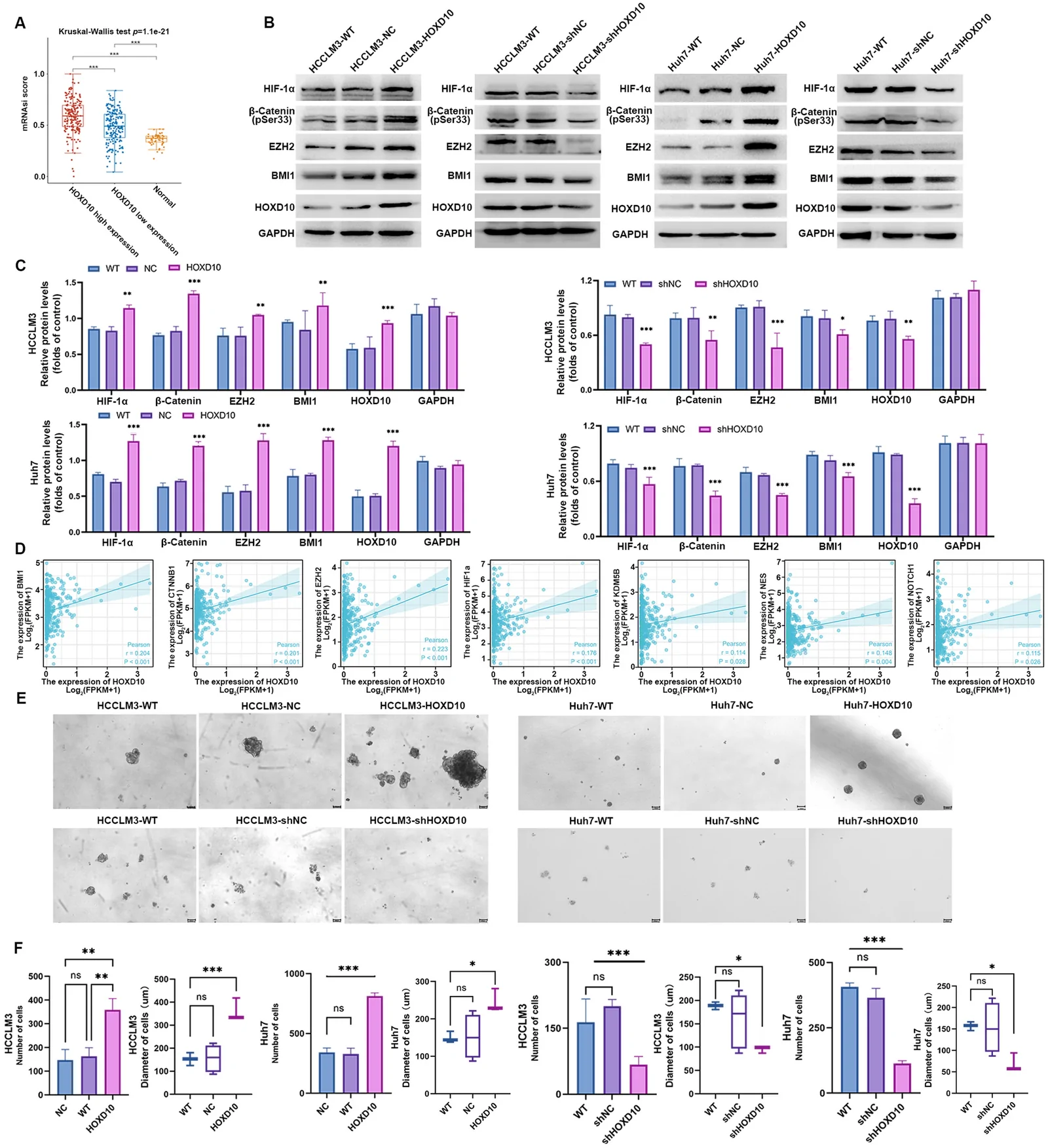
HOXD10 enhances cancer stem cell-like properties in HCC. (**A**) Stemness scores distribution among high, low, and normal HOXD10 expression groups from the TCGA database, with significant differences tested via the Kruskal-Wallis test. (**B**, **C**): Western blot analysis showing the correlation between HOXD10 expression and stemness-associated genes (HIF-1α, β-Catenin (pSer33), EZH2, and BMI1). Densitometry of western blot in panel. Data are mean ± SD (*n* = 3) and are representative of three independent experiments. Significance was determined using one-way ANOVA with Tukey’s multiple comparisons test. **p＜0.01, ***p＜0.001. (**D**) The relationship between HOXD10 expression and HCSC marker expression based on TCGA data. (**E**) Sphere formation assay demonstrating the differences in sphere-forming ability among HCC cells with different HOXD10 interventions. Scale bar: 200 μm. (**F**) Quantifying sphere count and size in HCCLM3 and Huh7 cells, data are shown as mean ± SD or median with interquartile range. Significance was assessed via one-way ANOVA with Tukey’s multiple comparisons test: *p < 0.05, **p < 0.01, ***p < 0.001, ns: not significant

To further elucidate the functional mechanisms of HOXD10, we assessed the stemness index of HCC samples utilizing the one-class logistic regression (OCLR) algorithm developed by Malta et al. [27]. The results of the rank-sum test indicated that the stemness index was significantly elevated in the group with high HOXD10 expression compared to both the low-expression and normal expression groups (Fig. 5A). Western blot analysis further revealed a positive correlation between HOXD10 expression and the expression levels of hepatocellular carcinoma stem cell (HCSC) markers, including hypoxia‑inducible factor 1α (HIF‑1α), β-Catenin (pSer33), enhancer of zeste 2 polycomb repressive complex 2 subunit (EZH2), and BMI1 proto-oncogene, polycomb ring finger (BMI1) (Fig. 5B, C). Consistent with these findings, Pearson correlation analysis of TCGA data also demonstrated a significant positive correlation between HOXD10 expression and HCSC marker expression (Fig. 5D). Moreover, sphere formation assays indicated that HOXD10 overexpression significantly enhanced the sphere-forming capacity of HCCLM3 and Huh7 cells, whereas HOXD10 silencing substantially inhibited this capacity (Fig. 5E, F). Overall, these findings suggested that the aberrant overexpression of HOXD10 facilitated self-renewal of HCSCs.

### HOXD10 activates the PI3K/AKT/mTOR signaling pathway

**Fig. 6:**
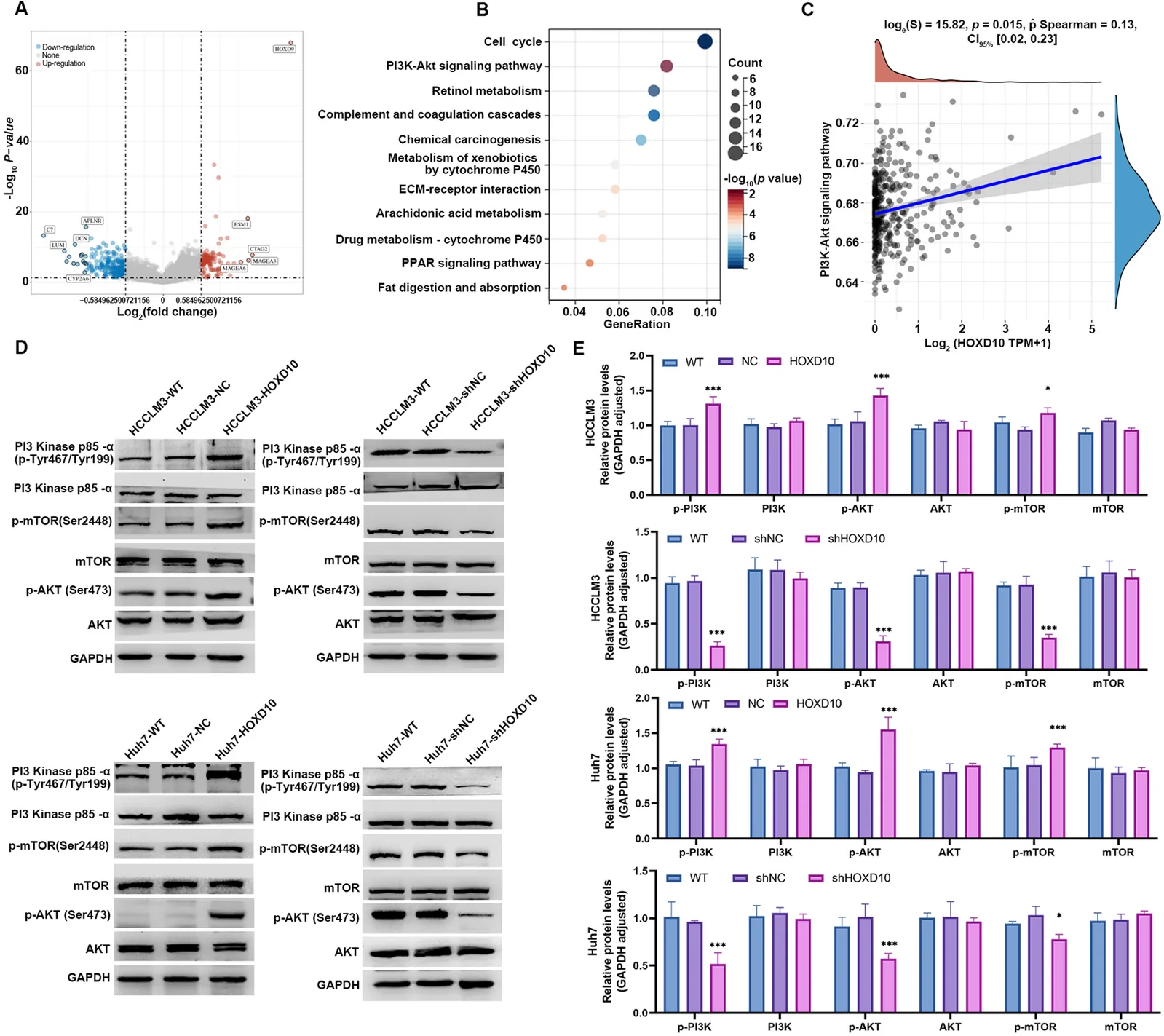
HOXD10 activates the PI3K/AKT/mTOR signaling pathway. (**A**) Volcano plot showing the differentially expressed genes (DEGs) between the HOXD10 high-expression and low-expression groups. (**B**) Bubble chart illustrating the KEGG pathways enriched by the DEGs, highlighting the PI3K/AKT/mTOR signaling pathway. (**C**) Scatter plot showing the positive correlation between HOXD10 expression and the PI3K/AKT/mTOR signaling pathway activity based on ssGSEA analysis. (**D**, **E**) Western blot analysis demonstrating the effects of altered HOXD10 expression on the phosphorylation levels of PI3K, AKT, and mTOR, indicating modulation of pathway activity. All data for the densitometric analysis of the western blots are presented as mean ± SD (*n* = 3) of three independent experiments. Significance was determined using two-way ANOVA with Dunnett’s multiple comparisons test. *p < 0.05, ***p < 0.001

To uncover the molecular processes through which HOXD10 influenced the progression of HCC, we conducted an analysis of TCGA data to identify differentially expressed genes (DEGs) between groups with high and low HOXD10 expression, utilizing the “Limma” package. This analysis identified 129 upregulated and 204 downregulated genes, with criteria of log2FoldChange ≥ 1.5 or ≤−1.5 and an adjusted *p*-value (padj) <0.05 (Fig. 6A). Subsequent KEGG pathway analysis indicated that these DEGs were enriched in 38 signaling pathways, with a false discovery rate (FDR) <0.01 and padj < 0.05 (Fig. 6B). Notably, the PI3K/AKT/mTOR signaling pathway emerged as a critical pathway in HCC progression. Aligned with these findings, single-sample ssGSEA demonstrated a significant positive correlation between HOXD10 expression and the activity of the PI3K/AKT/mTOR pathway (Fig. 6C). To validate this finding, western blot analyses were performed, which revealed that HOXD10 overexpression markedly enhanced the phosphorylation levels of PI3K, AKT, and mTOR, while HOXD10 knockdown resulted in a significant reduction in their phosphorylation (Fig. 6D, E). These results indicated that HOXD10 may regulate the biological behavior of HCC by modulating the PI3K/AKT/mTOR signaling pathway.

### HOXD10 expression modulates m6A regulatory gene expression

**Fig. 7:**
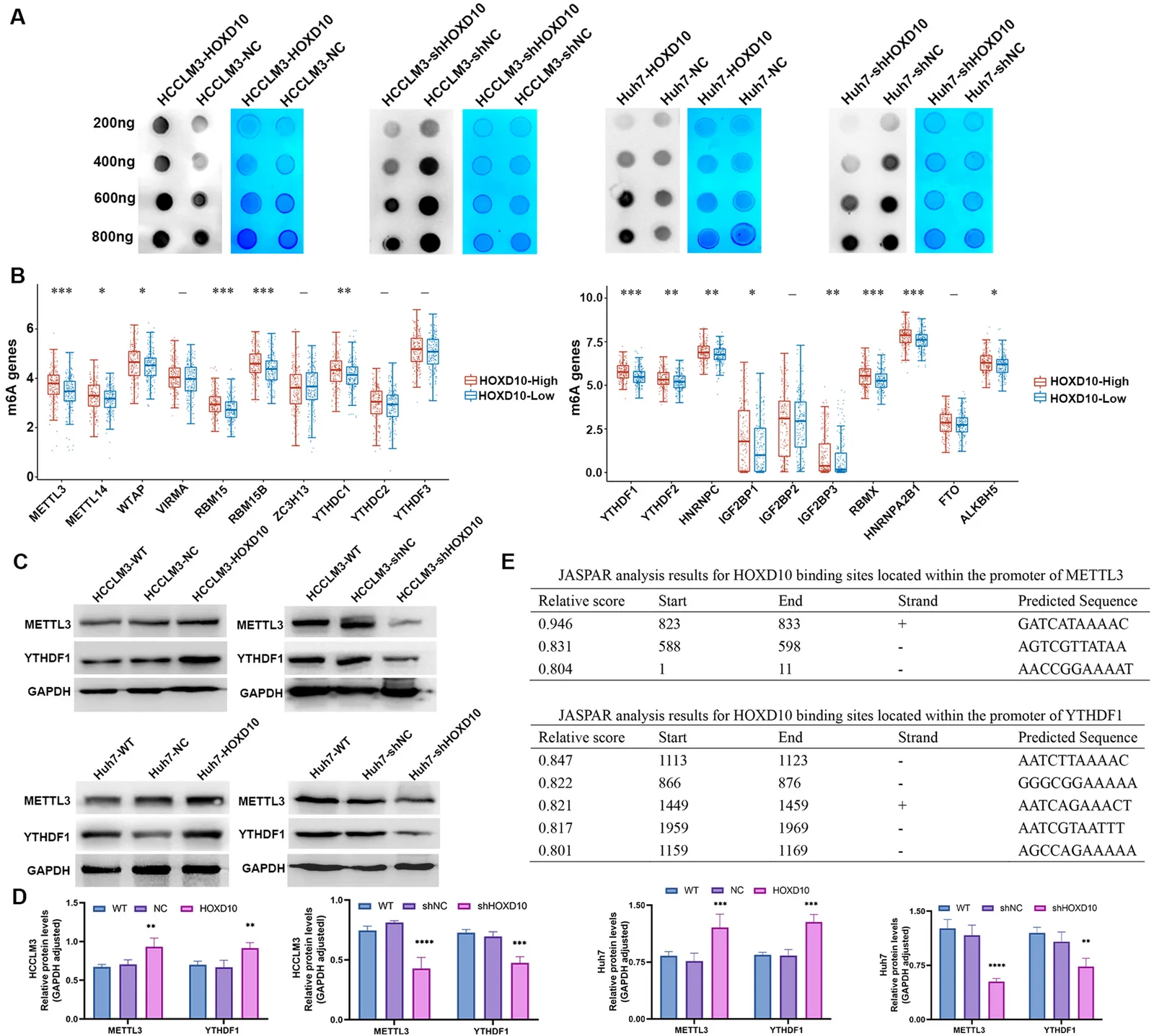
HOXD10 modulates m6A methylation and regulatory gene expression. (**A**) RNA dot blot assay illustrating the effect of altered HOXD10 expression on m6A methylation levels. (**B**) Differential expression analysis of m6A regulatory genes between HOXD10 high- and low-expression groups based on TCGA data. The significance of the results was assessed utilizing the Wilcoxon test. *p < 0.05, **p < 0.01, ***p < 0.001. (**C**, **D**) Western blot analysis showing the impact of HOXD10 expression changes on METTL3 and YTHDF1 protein levels. All data for the densitometric analysis of the western blots are presented as mean ± SD (*n* = 3) of three independent experiments. Significance was determined using two-way ANOVA with Dunnett’s multiple comparisons test. (**E**) JASPAR database prediction of potential HOXD10 binding sites in the promoter regions of METTL3 (3 sites) and YTHDF1 (5 sites)

To examine the influence of HOXD10 on m6A RNA methylation, we first employed an RNA dot blot assay to assess variations in m6A methylation levels across different experimental groups. The outcomes showed that m6A methylation levels were significantly elevated in the HOXD10 overexpression group compared to the control group, whereas the HOXD10 knockdown group demonstrated a marked reduction in m6A methylation levels (Fig. 7A). Subsequently, an analysis of TCGA data was conducted to assess the differential expression of known m6A regulatory genes between high and low HOXD10 expression groups, identifying 14 differentially expressed m6A regulators (Fig. 7B). Furthermore, we carried out Western blot analyses on two pivotal m6A regulatory proteins: the methyltransferase METTL3 and the m6A reader protein YTHDF1. The results indicated that HOXD10 overexpression enhanced the expression of METTL3 and YTHDF1, whereas HOXD10 knockdown suppressed their expression (Fig. 7C, D). Additionally, utilizing the JASPAR database [28], we predicted that the METTL3 promoter region contained three potential HOXD10 binding sites, while the YTHDF1 promoter region contained five potential HOXD10 binding sites (Fig. 7E). These findings suggested that HOXD10 may play a regulatory role in m6A RNA methylation.

### HOXD10-induced stemness and malignancy in HCC cells rely on METTL3 activating the PI3K/AKT/mTOR pathway

**Fig. 8:**
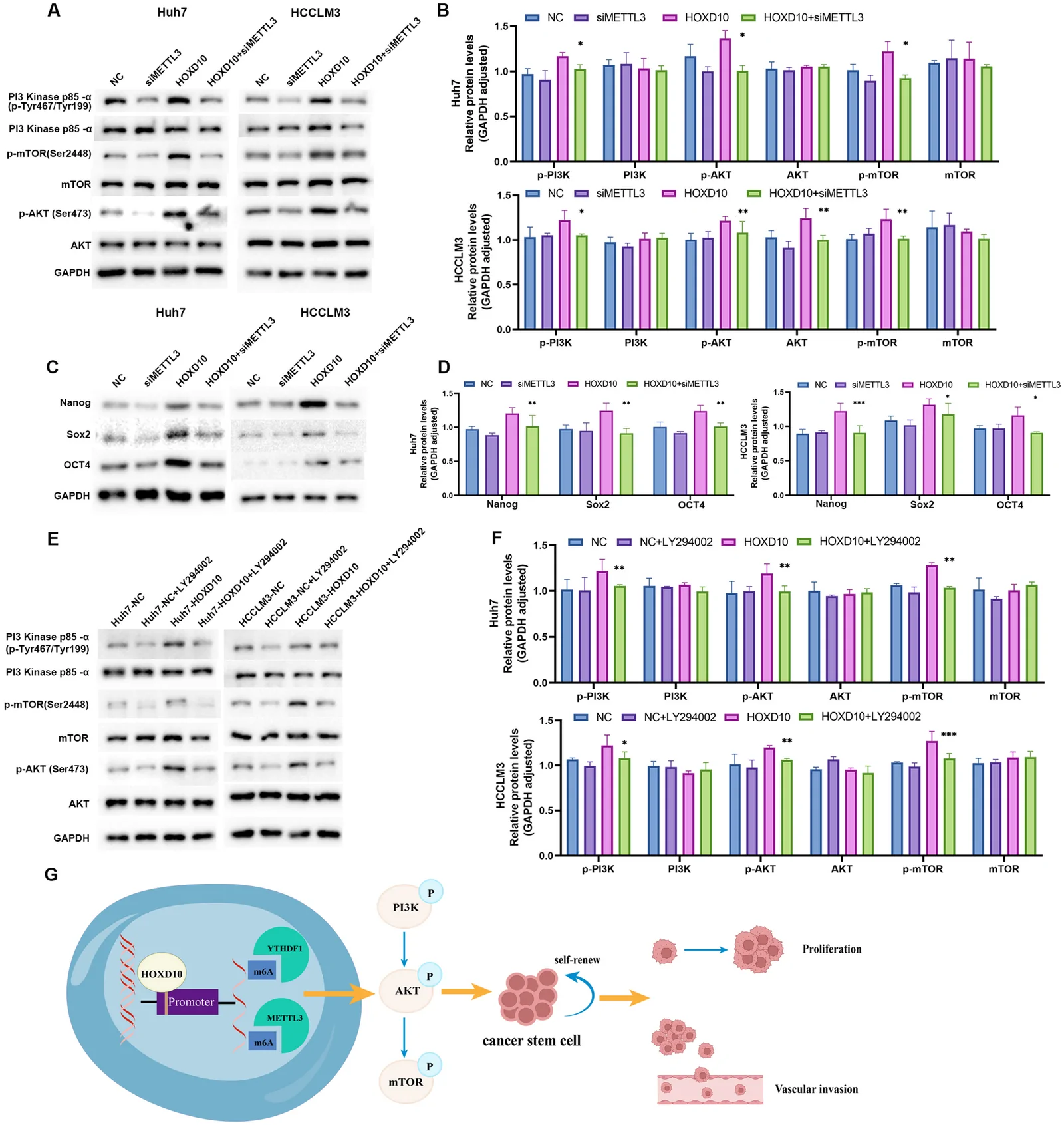
METTL3-mediated activation of the PI3K/AKT/mTOR pathway is required for HOXD10-induced stemness in HCC cells. (**A**, **B**) Western blot analysis of PI3K, p-PI3K, AKT, p-AKT, mTOR, and p-mTOR expression in HCC cells transfected with NC, siMETTL3, HOXD10, or HOXD10+siMETTL3. Representative blots and corresponding densitometric analyses are shown. Data are presented as mean ± SD (*n* = 3) of three independent experiments. Significance was determined using two-way ANOVA with Dunnett’s multiple comparisons test. (**C**, **D**) Western blot analysis of stemness-associated markers (NANOG, SOX2, and OCT4) in HCC cells under the indicated treatments. Representative blots and quantitative analyses are shown. Data are presented as mean ± SD (*n* = 3) of three independent experiments. Significance was determined using two-way ANOVA with Dunnett’s multiple comparisons test. (**E**, **F**) Western blot analysis of PI3K, p-PI3K, AKT, p-AKT, mTOR, and p-mTOR expression in HCC cells treated with NC, LY294002, HOXD10, or HOXD10+LY294002. Representative blots and quantitative analyses are shown. Data are presented as mean ± SD (*n* = 3) of three independent experiments. Significance was determined using two-way ANOVA with Dunnett’s multiple comparisons test. (**G**) A schematic representation illustrating the regulatory role of HOXD10 in the PI3K/AKT/mTOR signaling pathway via m6A methylation, which facilitates the self-renewal of HCSC and influences the progression of HCC. *p < 0.05, **p < 0.01, ***p < 0.001, ns, not significant

To explore the link between HOXD10, m6A methylation, and the PI3K/AKT/mTOR pathway, we silenced METTL3 with siRNA. Western blots showed that HOXD10 increased PI3K, AKT, and mTOR phosphorylation, but this effect was nullified by METTL3 knockdown (Fig. 8A, B). We also found that HOXD10 raised levels of cancer stem cell markers NANOG, SOX2, and OCT4, which METTL3 depletion reversed (Fig. 8C, D). Sphere formation assays confirmed that METTL3 is crucial for HOXD10 to maintain cancer stem cell-like traits in HCC cells, as METTL3 silencing reduced sphere number and size (Supplementary Fig. 2A, B). These findings confirm that METTL3 is essential for HOXD10 to sustain the cancer stem cell-like properties of HCC.

After overexpressing HOXD10, we used LY294002 to inhibit the PI3K/AKT/mTOR pathway. Western blot results showed that LY294002 reduced the phosphorylation levels of PI3K, AKT, and mTOR increased by HOXD10 (Fig. 8E, F). Further studies showed that inhibiting the PI3K/AKT/mTOR pathway with LY294002 reduced NANOG, SOX2, and OCT4 expression in HOXD10-overexpressing HCC cells (Supplementary Fig. 2C, D). Functional assays indicated that HOXD10 decreased apoptosis, but LY294002 reversed this effect (Supplementary Fig. 2E, F). Additionally, Transwell invasion assays confirmed that the enhancement of HCC cell invasion by HOXD10 was substantially diminished following the inhibition of the PI3K/AKT/mTOR pathway (Supplementary Fig. 2G, H).

We established a regulatory model (Fig. 8G) in which HOXD10 upregulates the expression of METTL3 and YTHDF1, thereby enhancing m6A RNA methylation. This molecular mechanism activates the PI3K/AKT/mTOR signaling pathway, which in turn facilitates the self-renewal and proliferation of hepatic cancer stem cells while inhibiting apoptosis. Consequently, these processes contribute to increased vascular invasion and tumor metastasis, ultimately driving the progression of hepatocellular carcinoma.

## Discussion

The molecular pathogenesis of HCC is characterized by complex regulatory networks, with accumulating evidence highlighting the pivotal role of the HOX gene family in hepatocarcinogenesis. Among them, HOXD10, a distinguished member of the HOX family, has emerged as a critical regulator in the initiation, progression, and metastasis of HCC [29]. Through a combination of clinical specimen analysis and functional experiments, our study demonstrated that overexpression of HOXD10 was significantly associated with aggressive tumor behavior, including advanced tumor stages (T3 and T4) and vascular invasion, as well as poor clinical outcomes in HCC patients. Crucially, HOXD10 is confirmed as an independent prognostic marker strongly associated with vascular invasion. These findings positioned HOXD10 as a promising therapeutic target for HCC, corroborating previous studies that explored the role of HOXD10 in various malignancies such as gastric and colorectal cancers [30–32].

HOXD10 shows context-dependent dual roles in different cancers. It often acts as a tumor suppressor in colorectal, ovarian, and gastric cancers due to promoter hypermethylation or miRNA modulation [30, 31, 33, 34]. However, in hepatocellular carcinoma (HCC), HOXD10 is upregulated, promoting cancer progression by increasing m6A methylation and activating the PI3K/AKT/mTOR pathway. These differences may result from unique epigenetic and regulatory factors in HCC. Clinically, high HOXD10 levels correlate with advanced tumor stages, vascular invasion, and poor survival, making it a potential prognostic biomarker and indicator of microvascular invasion.

In contrast to prior research [35, 36] which primarily focused on the role of HOXD10 in fundamental tumorigenic processes, our study offered novel insights into its function in promoting HCC cell self-renewal through modulation of stem cell-like characteristics. Notably, our findings underscored the regulatory role of HOXD10 in modulating the expression of METTL3 and YTHDF1, key regulators of m6A methylation, and activating the PI3K/AKT/mTOR pathway in HCC. We found that HOXD10 overexpression led to increased levels of METTL3 and YTHDF1, thereby enhancing m6A methylation. This, in turn, activated the PI3K/AKT/mTOR signaling pathway, driving HCC cell proliferation, migration, invasion, and the maintenance of stemness. Additional rescue assays confirmed the regulatory cascade, showing that silencing METTL3 or using the PI3K inhibitor LY294002 inhibited HOXD10‘s oncogenic activities, such as PI3K/AKT/mTOR signaling activation, stemness, invasion, and anti-apoptosis. This indicates that METTL3-mediated m6A methylation and the PI3K/AKT/mTOR pathway are crucial for HOXD10-driven HCC progression.

This new insight expanded our understanding of HOXD10‘s multifaceted role in HCC and aligned with previous studies linking HOX gene family members and PI3K/AKT signaling to VEGF-mediated angiogenesis, further emphasizing the interconnection of these pathways [37, 38]. Previous studies have established that both the PI3K/AKT/mTOR pathway and m6A methylation are crucial in tumor progression and angiogenesis [39]. Similar mechanisms have been observed in colorectal and gastric cancers [40, 41]. Specifically, the PI3K/AKT/mTOR pathway has been verified to upregulate HIF-1α expression, leading to increased VEGF production, which enhances tumor vascular permeability and facilitates tumor cell dissemination into the bloodstream, thus promoting metastasis [42]. Moreover, m6A methylation has been shown to enhance VEGF expression, further stimulating angiogenesis in colorectal cancer through the PI3K/AKT and ERK1/2 signaling pathways [43]. Li et al. [44] reported that m6A-mediated upregulation of HOXC10 contributed to HCC progression via the PTEN/AKT/mTOR pathway. Furthermore, m6A modifications and the HOX gene family have also been implicated in tumorigenesis and metastasis in gastric cancer and head and neck squamous cell carcinoma [24, 45]. In essence, our research provided a more comprehensive understanding of the molecular landscape of HCC, identifying potential therapeutic targets and laying the groundwork for future investigations aimed at developing more effective treatment strategies for this devastating disease.

Although conventional small-molecule drugs struggle to target transcription factors such as HOXD10, emerging proteolysis-targeting chimeras (PROTACs) offer a promising solution [46]. New TF-PROTAC technologies can target previously “undruggable” transcription factors by using E3 ubiquitin ligases for protein degradation [47]. Future research still needs to address several questions.To advance understanding and treatment of hepatic cancer, it’s crucial to validate HOXD10‘s binding to METTL3 and YTHDF1 promoters via ChIP-seq, and use MeRIP-seq to map the m6A transcriptome linked to cancer stem cell renewal and vasculogenic mimicry. Investigating m6A modification’s role in tumor immune evasion will elucidate HOXD10‘s impact on metastasis. Developing HOXD10-targeted PROTACs, alongside patient stratification and liquid biopsy, could turn these insights into precision therapies for HCC. Additionally, understanding the regulatory network involving HOXD10, m6A, and the PI3K/AKT/mTOR pathway is vital. Creating specific inhibitors for HOXD10 or its effectors and conducting preclinical and clinical trials are essential for assessing potential treatments for HCC.

## Conclusions

Our study identified HOXD10 as a critical regulator of HCC progression through its modulation of m6A RNA methylation and activation of the PI3K/AKT/mTOR signaling pathway. Elevated HOXD10 expression was strongly associated with poor prognosis and advanced disease stages, underscoring its potential as both a biomarker and therapeutic target. These findings significantly enhanced the understanding of HCC biology and paved the way for future research focused on developing innovative treatment strategies. Targeting HOXD10 and its associated pathways may offer substantial therapeutic advantages and improve clinical outcomes for patients with HCC.

## Electronic supplementary material

Below is the link to the electronic supplementary material.

Supplementary Material 1

Supplementary Material 2

Supplementary Material 3

Supplementary Material 4

## Data Availability

Not applicable.

## Abbreviations

AFP: Alpha-fetoprotein
ANLTs: Adjacent non-cancerous tissues
BSA: Bovine serum albumin
CCK-8: Cell Counting Kit-8
CSCs: Cancer stem cells
DAB: 3,3′-Diaminobenzidine
DCP: Des-γ-carboxy prothrombin
DFS: Disease-free survival
DMEM: Dulbecco’s Modified Eagle’s Medium
DEGs: Differentially expressed genes
EdU: 5-Ethynyl-2′-deoxyuridine
EZH2: Enhancer of zeste 2 polycomb repressive complex 2 subunit
FBS: Fetal bovine serum
FPKM: Fragments per kilobase million
FDR: False discovery rate
GDC: Genomic Data Commons
GO: Gene Ontology
HBV: Hepatitis B virus
HCV: Hepatitis C virus
HCC: Hepatocellular carcinoma
HCSCs: Hepatic cancer stem cells
HIF-1α: Hypoxia-inducible factor 1α
HOX: Homeobox
HOXD10: Homeobox D10
HRP: Horseradish peroxidase
ICGC: International Cancer Genome Consortium
IHC: Immunohistochemistry
IOD: Integrated optical density
KEGG: Kyoto Encyclopedia of Genes and Genomes
m6A: N6-Methyladenosine
METTL3: Methyltransferase-like 3
MCS: Multiple cloning site
OCLR: One-class logistic regression
OS: Overall survival
padj: Adjusted *p*-value
PBS: Phosphate-buffered saline
PCGs: Protein-coding genes
PI3K/AKT/mTOR: Phosphatidylinositol 3-kinase/Protein kinase B/Mammalian target of rapamycin
PFI: Progression-free interval
PVDF: Polyvinylidene fluoride
RIPA: Radio Immunoprecipitation Assay
SD: Standard Deviation
shRNA: Short hairpin RNA
ssGSEA: Single-sample Gene Set Enrichment Analysis
SDS-PAGE: Sodium dodecyl sulfate-polyacrylamide gel electrophoresis
TBST: Tris-buffered saline with Tween 20
TPM: Transcripts per million
TCGA: The Cancer Genome Atlas
VEGF: Vascular Endothelial Growth Factor
VM: Vasculogenic mimicry
YTHDF1 YTH: N6‑Methyladenosine RNA binding protein 1
p-β-Catenin (pSer33): Phosphorylated β-Catenin at Ser33
BMI1: BMI1 Proto-oncogene, polycomb ring finger
NANOG: Nanog homeobox
SOX2: SRY-box transcription factor 2
OCT4: Octamer-binding transcription factor 4
