# Supplementary material for: HOXD10 promotes hepatocellular carcinoma progression by enhancing m6A methylation and activating the PI3K/AKT/mTOR pathway
Source: J Transl Med. 2026 Jul 13;24:1261. doi: 10.1186/s12967-026-08632-x (PMC13647666; doi:10.1186/s12967-026-08632-x)
Supplement: Supplementary file 1 [file 12967_2026_8632_MOESM1_ESM.pdf]

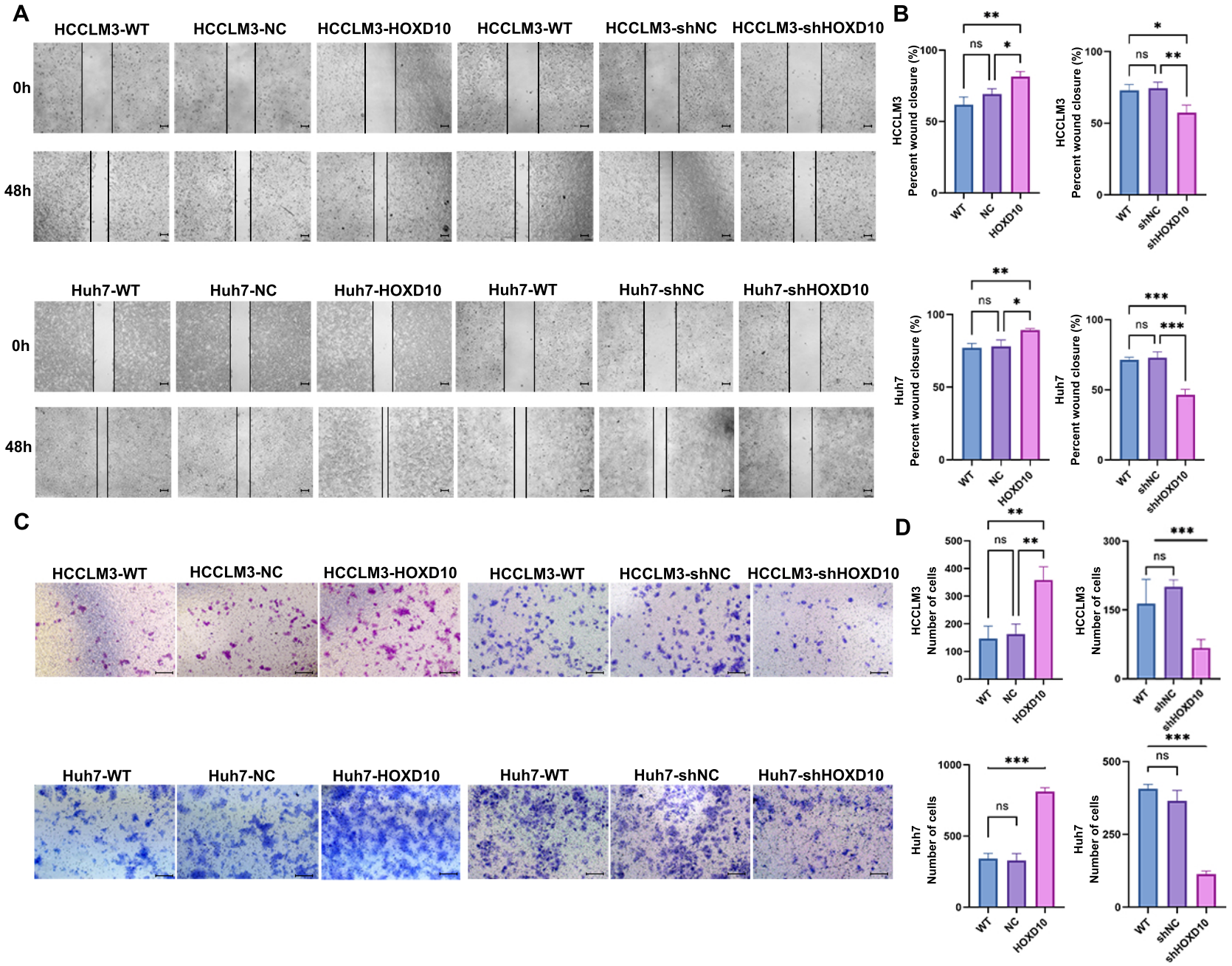

A
HCCLM3-WT
HCCLM3-NC
HCCLM3-HOXD10
HCCLM3-WT
HCCLM3-shNC
HCCLM3-shHOXD10
0h
48h
Huh7-WT
Huh7-NC
Huh7-HOXD10
Huh7-WT
Huh7-shNC
Huh7-shHOXD10
B
HCCLM3 Percent wound closure (%)
Huh7 Percent wound closure (%)
WT
NC
HOXD10
shNC
shHOXD10
ns
C
D
HCCLM3 Number of cells
Huh7 Number of cells
