# Supplementary material for: HOXD10 promotes hepatocellular carcinoma progression by enhancing m6A methylation and activating the PI3K/AKT/mTOR pathway
Source: J Transl Med. 2026 Jul 13;24:1261. doi: 10.1186/s12967-026-08632-x (PMC13647666; doi:10.1186/s12967-026-08632-x)
Supplement: Supplementary file 2 [file 12967_2026_8632_MOESM2_ESM.pdf]

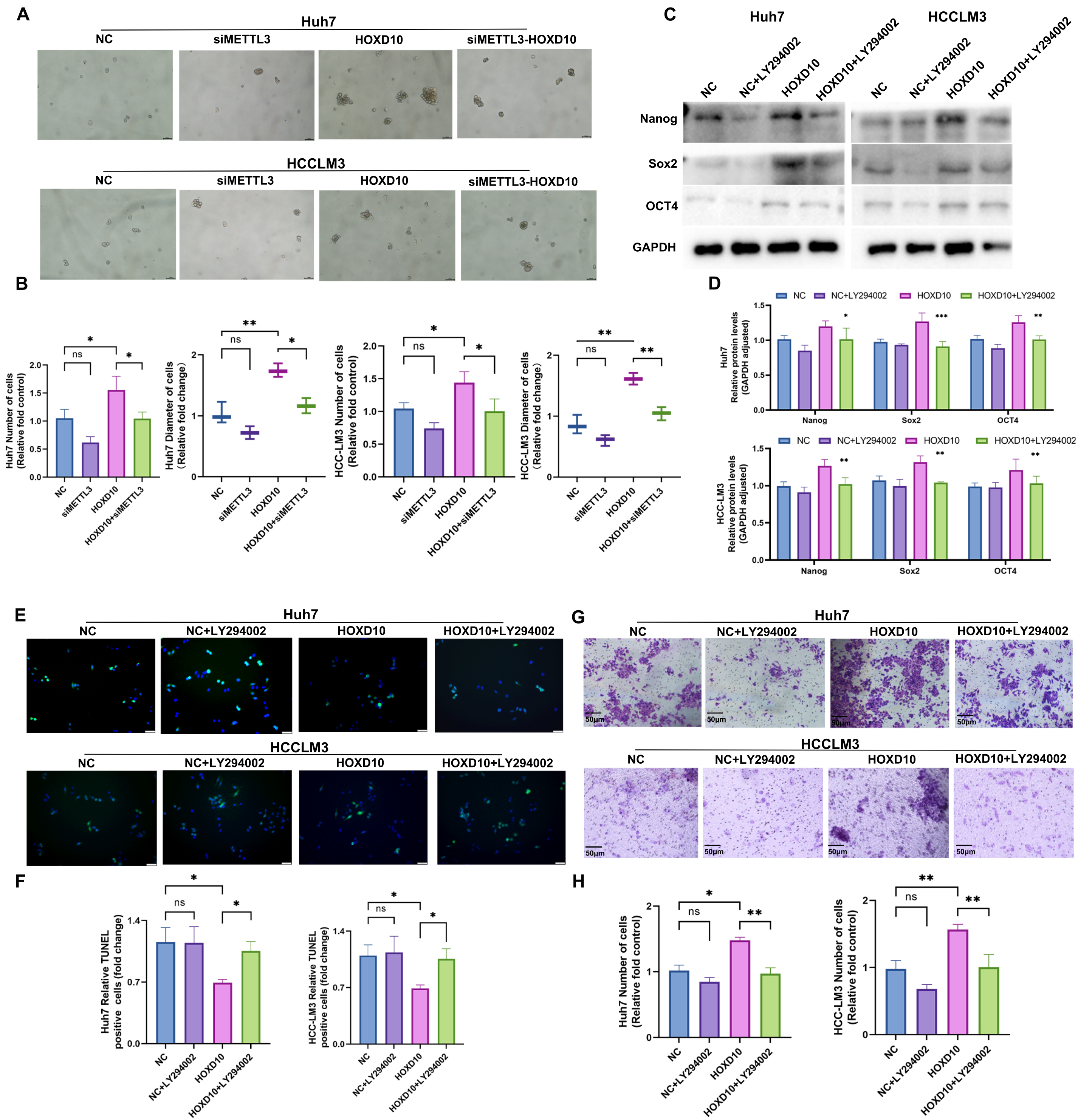

A
Huh7
NC
siMETTL3
HOXD10
siMETTL3-HOXD10
HCCLM3
NC
siMETTL3
HOXD10
siMETTL3-HOXD10
B
Huh7 Number of cells (Relative fold control)
Huh7 Diameter of cells (Relative fold change)
HCC-LM3 Number of cells (Relative fold control)
HCC-LM3 Diameter of cells (Relative fold change)
NC
siMETTL3
HOXD10
HOXD10+siMETTL3
ns
C
Huh7
HCCLM3
NC
NC+LY294002
HOXD10
HOXD10+LY294002
Nanog
Sox2
OCT4
GAPDH
D
NC
NC+LY294002
HOXD10
HOXD10+LY294002
Huh7 Relative protein levels (GAPDH adjusted)
HCC-LM3 Relative protein levels (GAPDH adjusted)
Nanog
Sox2
OCT4
E
Huh7
HCCLM3
NC
NC+LY294002
HOXD10
HOXD10+LY294002
F
Huh7 Relative TUNEL positive cells (fold change)
HCC-LM3 Relative TUNEL positive cells (fold change)
G
Huh7
HCCLM3
NC
NC+LY294002
HOXD10
HOXD10+LY294002
50μm
H
Huh7 Number of cells (Relative fold control)
HCC-LM3 Number of cells (Relative fold control)
NC
NC+LY294002
HOXD10
HOXD10+LY294002
ns
