# Supplementary material for: HOXD10 promotes hepatocellular carcinoma progression by enhancing m6A methylation and activating the PI3K/AKT/mTOR pathway
Source: J Transl Med. 2026 Jul 13;24:1261. doi: 10.1186/s12967-026-08632-x (PMC13647666; doi:10.1186/s12967-026-08632-x)
Supplement: Supplementary file 3 [file 12967_2026_8632_MOESM3_ESM.docx]

| Characteristics | Total (N) | Univariate analysis | |  | Multivariate analysis | |
| --- | --- | --- | --- | --- | --- | --- |
|  |  | Hazard ratio (95% CI) | P value |  | Hazard ratio (95% CI) | P value |
| Pathologic T stage | 370 |  |  |  |  |  |
| T1&T2 | 277 |  |  |  |  |  |
| T3&T4 | 93 | 2.598 (1.826 - 3.697) | **< 0.001** |  | 2.585 (1.664 - 4.016) | **< 0.001** |
| Pathologic N stage | 258 |  |  |  |  |  |
| N0 | 254 |  |  |  |  |  |
| N1 | 4 | 2.029 (0.497 - 8.281) | 0.324 |  |  |  |
| Pathologic M stage | 272 |  |  |  |  |  |
| M0 | 268 |  |  |  |  |  |
| M1 | 4 | 4.077 (1.281 - 12.973) | **0.017** |  | 2.325 (0.709 - 7.623) | 0.164 |
| Histologic grade | 368 |  |  |  |  |  |
| G1&G2 | 233 |  |  |  |  |  |
| G3&G4 | 135 | 1.091 (0.761 - 1.564) | 0.636 |  |  |  |
| AFP (ng/ml) | 279 |  |  |  |  |  |
| <= 400 | 215 |  |  |  |  |  |
| > 400 | 64 | 1.075 (0.658 - 1.759) | 0.772 |  |  |  |
| Vascular invasion | 317 |  |  |  |  |  |
| No | 208 |  |  |  |  |  |
| Yes | 109 | 1.344 (0.887 - 2.035) | 0.163 |  |  |  |
| HOXD10 | 373 |  |  |  |  |  |
| Low | 187 |  |  |  |  |  |
| High | 186 | 1.669 (1.175 - 2.370) | **0.004** |  | 1.597 (1.024 - 2.493) | **0.039** |

Supplementary Table 1: Cox Regression Analysis of Prognostic Factors for Overall Survival (OS) in HCC Patients.
