# Supplementary material for: HOXD10 promotes hepatocellular carcinoma progression by enhancing m6A methylation and activating the PI3K/AKT/mTOR pathway
Source: J Transl Med. 2026 Jul 13;24:1261. doi: 10.1186/s12967-026-08632-x (PMC13647666; doi:10.1186/s12967-026-08632-x)
Supplement: Supplementary file 4 [file 12967_2026_8632_MOESM4_ESM.docx]

| Characteristics | Total (N) | Univariate analysis | |  | Multivariate analysis | |
| --- | --- | --- | --- | --- | --- | --- |
|  |  | Hazard ratio (95% CI) | P value |  | Hazard ratio (95% CI) | P value |
| Pathologic T stage | 370 |  |  |  |  |  |
| T1&T2 | 277 |  |  |  |  |  |
| T3&T4 | 93 | 2.177 (1.590 - 2.980) | **< 0.001** |  | 1.461 (0.911 - 2.345) | 0.116 |
| Pathologic N stage | 258 |  |  |  |  |  |
| N0 | 254 |  |  |  |  |  |
| N1 | 4 | 1.370 (0.338 - 5.552) | 0.659 |  |  |  |
| Pathologic M stage | 272 |  |  |  |  |  |
| M0 | 268 |  |  |  |  |  |
| M1 | 4 | 3.476 (1.091 - 11.076) | **0.035** |  | 3.268 (0.975 - 10.961) | 0.055 |
| Histologic grade | 368 |  |  |  |  |  |
| G1&G2 | 233 |  |  |  |  |  |
| G3&G4 | 135 | 1.152 (0.853 - 1.557) | 0.355 |  |  |  |
| AFP (ng/ml) | 279 |  |  |  |  |  |
| <= 400 | 215 |  |  |  |  |  |
| > 400 | 64 | 1.045 (0.698 - 1.563) | 0.832 |  |  |  |
| Vascular invasion | 317 |  |  |  |  |  |
| No | 208 |  |  |  |  |  |
| Yes | 109 | 1.676 (1.196 - 2.348) | **0.003** |  | 1.281 (0.851 - 1.928) | 0.235 |
| HOXD10 | 373 |  |  |  |  |  |
| Low | 187 |  |  |  |  |  |
| High | 186 | 1.785 (1.330 - 2.396) | **< 0.001** |  | 1.679 (1.131 - 2.491) | **0.010** |

Supplementary Table 2: Cox Regression Analysis of Prognostic Factors for progression-free interval (PFI) in HCC Patients.
